# Th2/Th17 cell associated cytokines found in seroma fluids after breast cancer surgery

**DOI:** 10.1007/s00404-023-07074-w

**Published:** 2023-05-27

**Authors:** Nicole Pochert, Mariella Schneider, Melitta B. Köpke, Mathis Wild, Angelika Mattmer, Jacqueline Sagasser, Monika M. Golas, Maggie Banys-Paluchowski, Aline Metz, Christian Hinske, Matthias Reiger, Udo Jeschke, Christian Dannecker, Avidan Neumann, Claudia Traidl-Hoffmann, Michael Untch, Thorsten Kühn, Nina Ditsch

**Affiliations:** 1 Department of Obstetrics and Gynecology, University Hospital Augsburg, Augsburg, Germany; 2 Environmental Medicine, Faculty of Medicine, University of Augsburg, Augsburg, Germany; 3 Department of Data Management and Clinical Decision Support, Faculty of Medicine, University of Augsburg, Augsburg, Germany; 4 Human Genetics, Faculty of Medicine, University of Augsburg, Augsburg, Germany; 5 Department of Obstetrics and Gynecology, University Hospital Schleswig-Holstein, Campus Lübeck, Lübeck, Germany; 6 Department of Obstetrics and Gynecology, Helios Klinikum Berlin-Buch, Berlin, Germany; 7 Department of Obstetrics and Gynecology, Die Filderklinik, Filderstadt, Germany

**Keywords:** Breast cancer, Simple mastectomy, Seroma formation, Th2/Th17 immune response, Cytokines

## Abstract

**Purpose:**

The development of a seroma after breast cancer surgery is a common postoperative complication seen after simple mastectomy and axillary surgery. We could recently demonstrate that breast cancer patients undergoing a simple mastectomy with subsequent seroma formation developed a T-helper cell increase within the aspirated fluid measured by flow cytometry. The same study revealed a Th2 and/or a Th17 immune response in peripheral blood and seroma fluid of the same patient. Based on these results and within the same study population, we now analyzed the Th2/Th17 cell associated cytokine content as well as the best known clinical important cytokine IL-6.

**Methods:**

Multiplex cytokine measurements (IL-4, IL-5, IL-13, IL-10, IL-17, and IL-22) were done on 34 seroma fluids (Sf) after fine needle aspiration of patients who developed a seroma after a simple mastectomy. Serum of the same patient (Sp) and that of healthy volunteers (Sc) were used as controls.

**Results:**

We found the Sf to be highly cytokine rich. Almost all analyzed cytokines were significantly higher in abundance in the Sf compared to Sp and Sc, especially IL-6, which promotes Th17 differentiation as well as suppresses Th1 differentiation in favor of Th2 development.

**Conclusion:**

Our Sf cytokine measurements reflect a local immune event. In contrast, former study results on T-helper cell populations in both Sf and Sp tend to demonstrate a systemic immune process.

## What does this study add to the clinical work

**Table:** 

Seroma formation after breast surgery is associated with an inflammatory immune response resulting in an increase of Th2/17 associated cytokines.	

## Introduction

The formation of a seroma is a very common postoperative complication seen after breast and axillary surgery, especially in case of a mastectomy [1–4]. To avoid seroma formation, the origin and composition must first be understood, to develop avoidance strategies. The current level of knowledge remains low: seroma formation is defined as a subcutaneous accumulation of fluid, of which the origin is unknown and still controversially discussed. The fluid collection was originally considered as a result of a pure lymphatic obstruction, whereas other studies show characteristics of an exudate [5–8]. Comparing all of those studies, the time point of fluid analysis seems to be of high importance.

A recent study by our group analyzed the cell content of the seroma fluids and found a specific immunological response through certain T-helper (Th) cell subpopulations. Significantly higher numbers of Th2 and Th17 cells were found in seroma fluid and peripheral blood of the same patients compared to peripheral blood of healthy controls [9]. As known from literature, activated Th cells can differentiate into several functional classes based on their surrounding environment, namely antigen presenting cells (APCs), cytokines, and other costimulatory molecules [10]. Cytokines are known for their immunomodulatory capabilities and are a large and diverse family of small proteins or glycoproteins. Additionally, it was shown that they play a role in developmental processes separate from the immune system, such as cell differentiation and directed migration. Therefore, influencing both, innate and adaptive immune responses. Cytokines are mainly produced by Th cells or macrophages, although they can be transiently induced and secreted by virtually all nucleated cells [11]. In our first publication on seroma immune cell composition, we were able to differentiate Th2/Th17 cells by flow cytometry in seroma fluid (Sf) and peripheral blood, but the measurement of specific cytokines allows a broader picture of T-helper cell differentiation found in Sf.

The Th1/Th2 cell subclass model was discovered based on differential cytokine production and surface marker expression in the 1980s [12]. The Th1/Th2 cell model has many inconsistencies, because human cytokines are rarely exclusive pro-Th1 or -Th2. Therefore, a modern multiplex cytokine measurement approach allows to identify a broad spectrum of cytokines on one hand but also relate these cytokines to Th1 or Th2 cell compositions, as well as to the Th9, Th17, and Th22 cell model, developed decades later [13, 14]. Differentiation into Th2 effector cells is dependent on the presence of Interleukin (IL)-4 [15, 16]. A positive-feedback loop is supported by autocrine IL-4 secretion of Th2 cells, and also they express IL-5 and IL-13 as their signature cytokines [16]. IL-4 combined with IL-6 from APCs suppress Th1 differentiation and modulate the Th1/Th2 balance toward Th2 [15–19]. Protective immune responses orchestrated by Th2 cells aim to target helminths and facilitate tissue repair, but can also contribute to chronic inflammatory diseases, including asthma and allergy [20]. Besides others, Th17 cells are described by the production of IL-10, IL-17, and IL-22 [10]. Polarization proceeds through TGFβ and IL-6, and autocrine amplification by IL-21 production and secretion of IL-23 from APCs stabilizes the Th17 lineage [15, 16, 18].

Based on the lack of scientific knowledge about seroma formation after breast cancer surgery, there is a demand for unraveling the underlying mechanisms of seroma formation for early detection, possible prevention, and/or better treatment strategies. Therefore, one of the aims of the pilot phase of our study was to analyze the cytokine content of collected Sf in patients with simple mastectomy after fine needle aspiration with the help of a multiplex cytokine approach. Simultaneously cytokine levels have been measured in serum of seroma patients (Sp) as well as serum of the healthy controls (Sc). Another aim of this study was to compare certain cytokines measured in this study with the specific Th2/Th17 cell response found in Sf in our former study [9].

## Materials and methods

### Patients

A first interim analysis of the monocentric pilot study of the international multicenter SerMa study planned to start in July 2023 (EUBREAST 5), where the study concept was validated, included a subgroup population of patients with a surgical procedure of a simple mastectomy with or without sentinel lymph-node excision (SLNE) or/and axillary lymph-node dissection (ALND) and development of a puncturable seroma. SLNE was only performed in patients with invasive disease, cN0 (clinically not affected axillary lymph nodes) and DCIS due to the indication for a mastectomy. All patients presented with invasive breast cancer or DCIS. 34 seroma samples were collected using needle aspiration. In five patients, more than one fine needle aspiration was performed. All the patients gave an informed consent and an ethical committee permitted the study.

### Sample processing

Sf and Sp as well as Sc were transported to the laboratory and processed within 24 h. After centrifugation of the Sf, the supernatant was carefully removed from the cell pellet and stored at − 80 ℃. The serum monovettes from patients and controls were centrifuged for 10 min at room temperature, and the serum was carefully removed and stored at − 80 ℃.

### Cytokine assays

A “Bio-Plex Pro human Cytokine Screening Panel 48 plex” was used to determine the concentrations of 48 cytokines according to the manufacturer’s instructions (BioRad, Feldkirchen, Germany). Based on our former flow cytometry results, we analyzed and focused on the Th2 cytokines (IL-4, IL-5, and IL-13), Th17 cytokines (IL-6, IL-17, and IL-10), and since it was not included in the Bio-Plex Pro human Cytokine Screening Panel, the Th17 cytokine IL-22. The latter was determined using a DuoSet ELISA kit (R&D Systems) according to the manufacturer’s instructions. Undiluted samples were used and the absorbance was measured at 450 nm with a plate reader (Tecan Spark, Männedorf, Switzerland). Results were evaluated by comparing the measured absorbance after blank reduction to a standard curve provided in the kit.

### Statistical analysis

Statistical and correlation analysis was performed using GraphPad Prism. Results were analyzed by either a one-way-ANOVA or Kruskal–Wallis test. A value of *p* < 0.05 was considered statistically significant.

## Results

### Patient cohort

A subset of 16 patients treated between 10/2020 and 09/2021 and fulfilling the inclusion criteria (two with bilateral disease) were selected. Simple mastectomy was performed in all of the patients, also in cases with very small invasive tumors and a large concomitant DCIS. SLNE was performed in 12 patients (two both sided) including one DCIS due to the indication to mastectomy. ALND was done in 5 cases (one after SLNE and four due to multiple pathological lymph nodes). Median age was 73 (48–85). Seven of those patients had neoadjuvant chemotherapy. Radiation and endocrine therapy have been done following the general guideline recommendations [21]. The cohort of healthy volunteers consisted of 15 women with a median age of 38 (20–54). Tumor characteristics of the study population are described in Table 1.

**Table 1:** Description of tumor characteristics of 16 patients and 18 tumors (including two bilateral)

Tumor characteristics	Subgroups	*n* (number)
*Histopathological type*		
	NST Invasive lobular *Mucinous* *Apocrine* *Only DCIS* *Solid papillary*	11 3 1 1 1 1
*Focality* (2 bilateral carcinomas)		
	Unifocal Multifocal Multicentric	12 2 4
*Hormone receptor status* (without DCIS, 2 bilateral carcinomas)		
	HR + ER-/PR-	14 3
*Her-2/neu* (without DCIS; 2 bilateral carcinomas)		
	Positive Negative	2 15
*Ki67* (without DCIS; 2 bilateral carcinomas)		
	< 20% ≥ 20%	8 9
*Tumor size* (2 bilateral carcinomas)		
	ypT0 pTis (y)pT1a (y)pT1b (y)pT1c (y)pT2 (y)pT3 (y)pT4	3 1 1 2 2 5 4 0
*Axillary nodal status* (2 bilateral carcinomas)		
	(y)pN0 (y)pN1 (y)pN2	13 3 2
*Grading* (without DCIS; 2 bilateral carcinomas)		
	G1 G2 G3	2 11 4

### Cytokine profile in Sf and serum

**Fig. 1:**
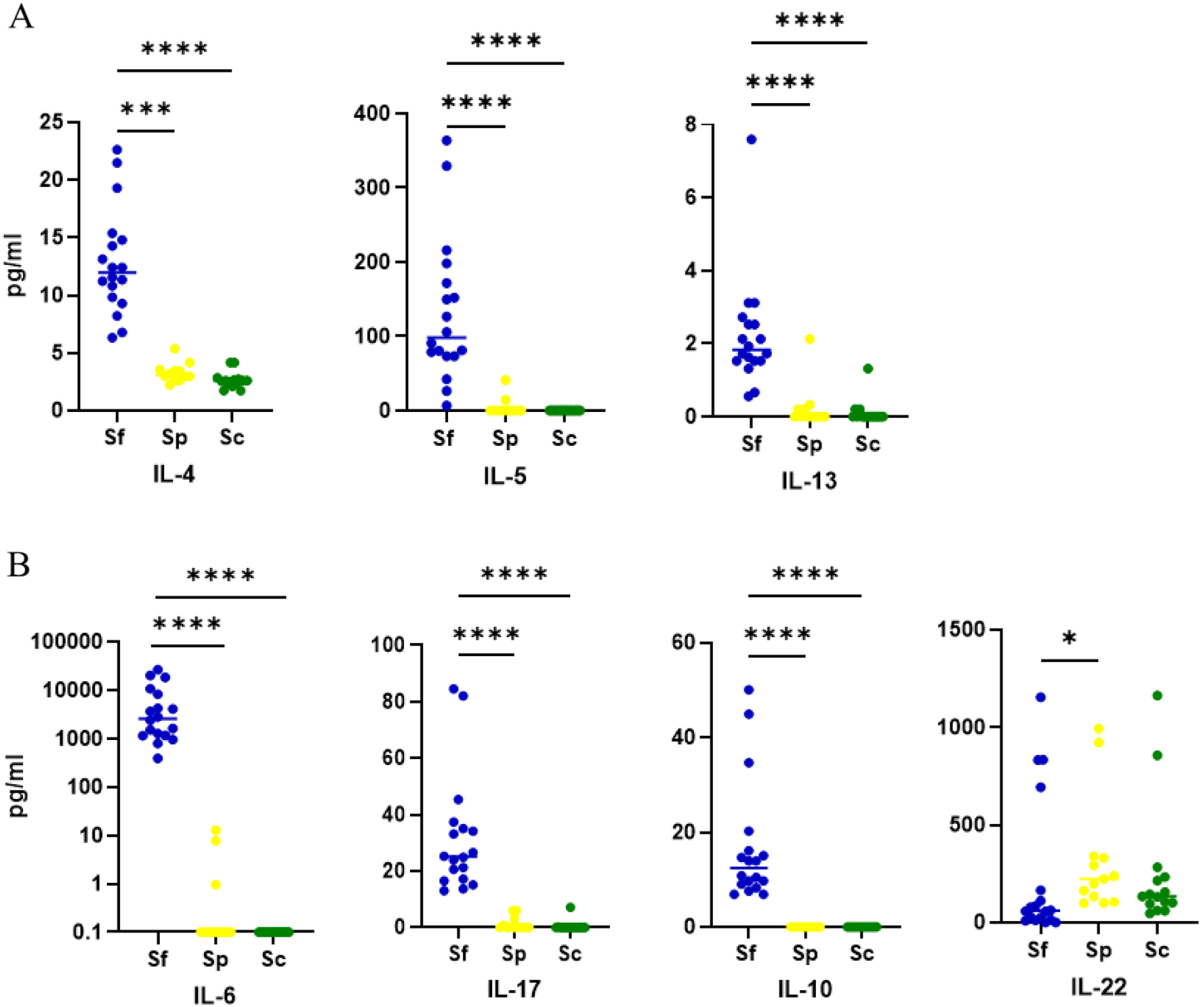
Cytokine level in pg/ml measured with the Bio-Plex platform or ELISA. **A** Cytokine important for the differentiation of Th2 cells, IL-4, and Th2 cell-secreted cytokines IL-5 and IL-13 in pg/ml. **B** Cytokine important for the differentiation of Th17 cells, IL-6, and cytokines secreted by Th17 cells: IL-17, IL-10, and IL-22 in pg/ml. *p < 0.05, ***p < 0.001, ****p < 0.0001

The Th2 differentiation-related cytokine IL-4 was detected in significantly higher amounts in Sf (12.84 ± 4.59 pg/ml) compared to Sp (3.3 ± 0.83 pg/ml) and Sc (2.65 ± 0.7 pg/ml). In addition to IL-4, mature Th2 cells secrete IL-5 and IL-13. We found significantly higher amounts of IL-5 in Sf (131.5 ± 96.6 pg/ml) compared to serum were IL-5 was nearly absent (Sp: 4.6 ± 12.2 pg/ml; Sc: 0.0 pg/ml). IL-13 levels show the same pattern, although total amounts in Sf (2.2 ± 1.5 pg/ml; Sp: 0.2 ± 0.6 pg/ml; Sc: 0.1 ± 0.3 pg/ml) were lower compared to IL-5 (Fig. 1A).

One important cytokine for the differentiation of naïve Th cells into Th17 cells is IL-6. We measured up to 10.000 fold higher levels of IL-6 in Sf (6.2 ± 7.9 ng/ml), which is highly significant compared to Sp (1.9 ± 4.1 pg/ml) and Sc (0.1 ± 0 pg/ml), where IL-6 is not detectable in most samples (Fig. 1B). Cytokines secreted by Th17 cells, IL-17, and IL-10 have also been found in significantly higher levels in Sf than in serum (Sp and Sc). In contrast, IL-22, also a secretion product of Th17 cells, was found in significantly lower levels in Sf (234.6 ± 365.9 pg/ml) compared to Sp (318.7 ± 295.9 pg/ml). Compared to Sc (252.8 ± 319.7 pg/ml), we could not see a significant difference.

### Comparative analyses of cytokine measurements and cellular content of Th2/Th17 cells

**Fig. 2:**
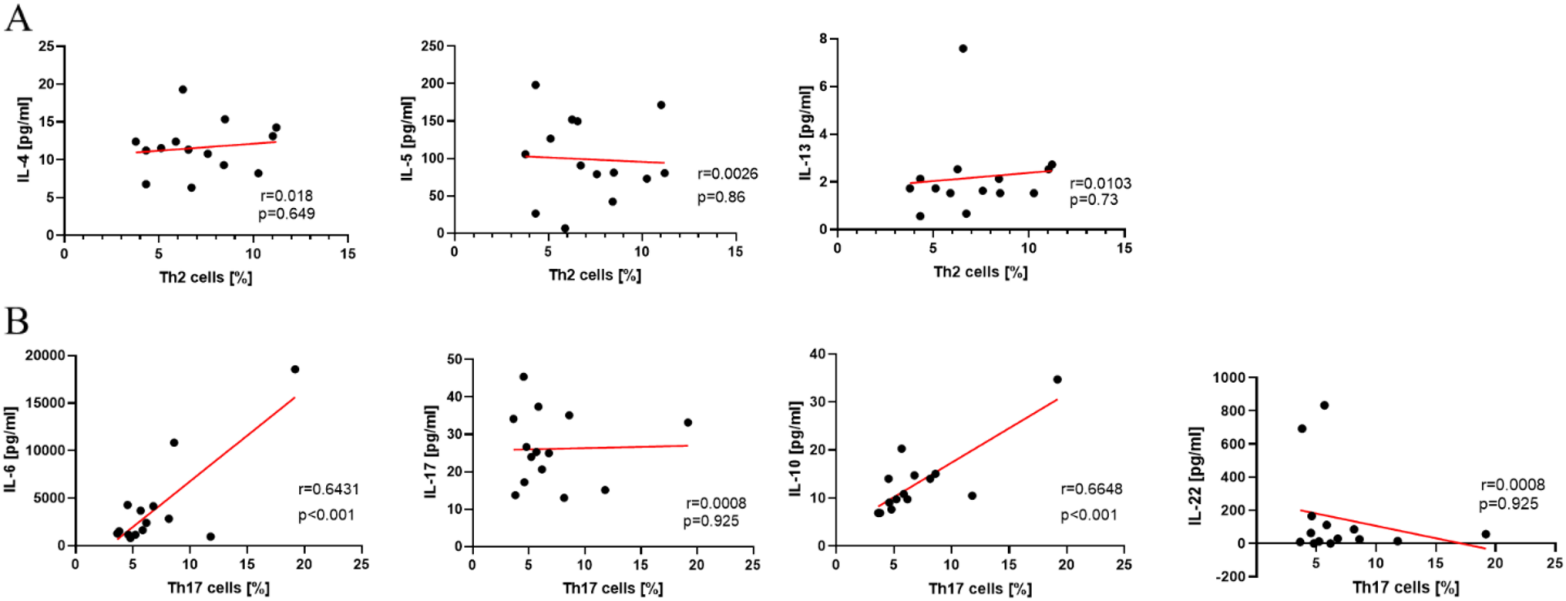
Correlation analysis between cytokines and number of Th2/Th17 cells in Sf. **A** Correlation between number of Th2 cells [%] and IL-4, IL-5, and IL-13 levels [pg/ml]. No significant correlation was found. **B** Correlation analysis between Th17 cells [%] and IL-6, IL-17, IL-17, and IL-22 levels [pg/ml]. Significant correlations were found for IL-6 (***) and IL-10 (***). ***p < 0.001

Within this study, we focused on Th2 and Th17 cytokines. Based on the results of our former flow cytometry analyses of the cellular Sf contend, we were able to compare both measurements. We found a highly significant correlation between the levels of IL-6 as well as IL-10 and the number of Th17 cells (Fig. 2). All other correlations were not significant.

## Discussion

These are the first published results of the pilot study of the SerMa study (EUBREAST 5), describing the cytokine content in relation to the Th-cellular content of seroma fluids in our former publication [9]. We elucidated connections between the specific Th2/Th17 immune response by analyzing the cytokine abundance of the collected fluids in comparison to the cellular content. In our former study, we found significantly higher percentages of Th2 cells in seroma fluid and peripheral blood of the patients compared to blood of healthy controls [9]. These results are now supported by the findings of the associated ILs, especially the significantly higher levels of IL-4, the most important cytokine regarding Th2 differentiation, measured in Sf. It is known that B cells, NKT cells, naïve Th cells, and mast cells can secrete IL-4 and, therefore, promote Th2 differentiation [22]. Interestingly, we found significantly lower numbers of B cells, NKT, and naïve Th cells in Sf compared to peripheral blood of patients and healthy controls, excluding those as the origin of IL-4 found in Sf [9]. Therefore, mast cells could potentially be a source of IL-4 in Sf. Additionally, Th2 cells support a positive-feedback loop by an autocrine secretion of IL-4 [16]. Significantly higher Th2 cell percentages in Sf and blood of patients could also be due to the very high levels of IL-6 measured in Sf. We also measured this cytokine in Sf. IL-4 in combination with IL-6 suppresses Th1 differentiation in favor toward Th2 development [15–19]. These increased IL-4 and IL-6 levels support our finding of a strong Th2 cell response in Sf.

In addition, IL-6 is an essential differentiation factor for Th17 cells [23, 24]. There were significantly more Th17 cells in Sf and blood compared to healthy controls [9]. In addition, we performed a correlation analysis between flow cytometry results and Bio-Plex cytokine measurements in Sf. IL-6 is highly abundant in Sf, and we could show a significant positive correlation between Th17 cell percentage and levels of IL-6 in the collected fluids, suggesting a direct connection between IL-6 levels and Th17 cell abundance. For the cytokines secreted by Th17 cells, we measured significantly higher IL-17 amounts in Sf. Interestingly, we found a positive correlation between Th17 cells and the abundance of IL-10, suggesting that most of the Th17 cells found in the Sf secrete IL-10 into their surroundings. It has been shown that different cytokines promoting Th17 differentiation can induce unique types of Th17 cells. TGF-β induces an IL-10-producing and less pathogenic subtype, while IL-1β promotes a more pro-inflammatory type of Th17 cells [16, 25–27]. These results verify the previous flow cytometry results by simple Bio-Plex cytokine measurements.

IL-4 serum levels in healthy individuals and in patients did not differ, indicating this cytokine as a rather local immune marker in contrast to Th2 cell, which showed a systemic abundance [9]. There is a study, which showed a global decrease in cytokine serum levels after neoadjuvant chemotherapy [28]. This effect we could not see in serum of our participants who received a neoadjuvant chemotherapy compared to healthy controls. Even though the median age of the control group is lower than our participants. There is evidence that serum cytokine levels could change with age, but for IL-6 and Ll-17, no differences could be found regarding age [29]. IL-6 levels are very high in Sf, but not detectable in Sp and cannot be connected to the higher numbers of Th17 cells found in peripheral blood of patients compared to healthy controls. Additionally, there was no direct correlation between IL-17 levels and Th17 cells numbers in Sf. All these results indicate a locally restricted immune response regarding cytokines rather than a systemic change as seen with the Th2/Th17 cells. These findings must be interpreted with caution in light of the limited number of patient cases and therefore have to be verified in a larger collective.

In summary, we could show that Sf is highly cytokine rich. Whereas, flow cytometry analysis of Th cell subpopulations showed a significantly higher cell abundance in seroma formations as well as peripheral blood of patients compared to healthy controls and, therefore, are rather connected to systemic processes. We found significant cytokine concentration changes in Sf but not in Sp or Sc. Therefore, cytokines were found to be rather locally constricted at the site of seroma formation and not systemically in serum. Further investigations will lead to more precise statements within the prospectively planned international multicenter SerMa study (EUBREAST5).

## Conclusions

This study demonstrated a successful determination of different cytokines in postoperatively developed seromas fluids of patients diagnosed with breast cancer and are locally treated with a simple mastectomy with or without an axillary surgical procedure. The Th2 differentiation-related cytokine IL-4 was found in significantly higher concentrations in Sf compared to serum (Sp and Sc). Additionally, we measured very high levels of IL-6 in Sf, also highly significant compared to Sp and Sc. IL-6 is known to promote Th17 differentiation as well as suppressing Th1 differentiation in favor toward Th2 development. Therefore, we were able to explain a concordance of cytokine expression and Th cell differentiation in seroma patients. High cytokine levels in Sf and low in Sp and Sc support a local limited immunological event in seromas.

## Data Availability

Additional data are available from the authors upon request based on ethical requirements.
